# Development of Bioadhesive Chitosan Superporous Hydrogel Composite Particles Based Intestinal Drug Delivery System

**DOI:** 10.1155/2013/563651

**Published:** 2013-08-04

**Authors:** Hitesh Chavda, Ishan Modhia, Anant Mehta, Rupal Patel, Chhagan Patel

**Affiliations:** ^1^Department of Pharmaceutics and Pharmaceutical Technology, Shri Sarvajanik Pharmacy College, Gujarat Technological University, Mehsana, Gujarat 384 001, India; ^2^Department of Pharmaceutical Chemistry, Shri Sarvajanik Pharmacy College, Gujarat Technological University, Mehsana, Gujarat 384 001, India

## Abstract

Bioadhesive superporous hydrogel composite (SPHC) particles were developed for an intestinal delivery of metoprolol succinate and characterized for density, porosity, swelling, morphology, and bioadhesion studies. Chitosan and HPMC were used as bioadhesive and release retardant polymers, respectively. A 3^2^ full factorial design was applied to optimize the concentration of chitosan and HPMC. The drug loaded bioadhesive SPHC particles were filled in capsule, and the capsule was coated with cellulose acetate phthalate and evaluated for drug content, *in vitro* drug release, and stability studies. To ascertain the drug release kinetics, the drug release profiles were fitted for mathematical models. The prepared system remains bioadhesive up to eight hours in intestine and showed Hixson-Crowell release with anomalous nonfickian type of drug transport. The application of SPHC polymer particles as a biomaterial carrier opens a new insight into bioadhesive drug delivery system and could be a future platform for other molecules for intestinal delivery.

## 1. Introduction

Hydrogels are polymers that will swell when placed in water and maintain their shape. There has been much research on the use of hydrogels in the medical field and, in particular, controlled drug delivery. Hydrogels can be formed by polymerization of hydrophilic monomers in the presence of cross-linker, or by cross-linking existing hydrophilic polymer chains. SPHs are hydrogels with highly (super-) porous structure with pore size between 100 *μ*m and 1000 *μ*m. They are commonly prepared by the gas blowing method. SPHs are prepared by cross-linking of monomer solution using chemical compounds in the presence of gas bubbles. When SPH forms, the polymer chains are separated by numerous pores. The pores of SPHs were all connected to form capillary channels so that water could be absorbed into the hydrogels by capillary action. The porous structures also offered SPHs with hundreds of times more surface area and shorter diffusion distance than those of conventional hydrogels. Consequently, the swelling kinetics of SPHs was hundred times faster than that of conventional hydrogels [1, 2]. SPH absorbs water very rapidly and swells to equilibrium size in a short period of time [3–7]. The second method uses irradiation for cross-linking of linear polymers [8]. Several important properties of SPHs, such as fast swelling, large swelling ratio, and surface slipperiness, make them excellent candidate materials to develop drug delivery devices [9]. Controlled delivery systems for the drug octreotide have been developed using SPH and SPH composite (SPHC) polymers, which were able to swell very quickly due to their highly porous structure, that kept the delivery system mechanically attached to the intestinal mucosa [10, 11].

Chitosan is a natural and biologically safe polymer. It is synthesized from chitin by the process of deacetylation. Chitosan and its derivatives are widely used as oral drug delivery vehicles as they are biocompatible, biodegradable and nontoxic [12–14]. Hydroxypropyl-methylcellulose (HPMC) is also the most widely used polymers in the preparation of oral controlled drug delivery systems. HPMC provides controlled release once it hydrates to form a gelatinous layer which controls the water transport in the system [15].

Microparticles constitute a major part of particulate drug delivery systems due to their small size and efficient carrier capacity. Unfortunately, microparticles have shorter residence time at site of absorption and so the coupling of bioadhesion characteristics would be more beneficial for prolonged contact with mucosal membrane. Bioadhesive microspheres include microparticles and microcapsules. Bioadhesion provides efficient absorption, enhanced bioavailability, more intimate contact with the mucosal layer, and site specific targeting of drug to the absorption site [16–19].

 Dorkoosh et al. [20] studied the cytotoxic effects of SPHs and SPHCs using propidium iodide staining, MTT assay, and trypan blue test. It was observed that than 95% of cells were viable after incubation with SPHCs. Yin et al. [21] and Tang et al. [22] have shown that the SPHCs were considered to be biocompatible and a safe carrier for peptide and protein drugs. It also showed low amount of residual solvents indicating good biocompatibility. Risbud and Bhonde [23] reported that hydrogels of polyacrylamide-chitosan were biocompatible, and no deleterious effects of on cell viability and functionality, as there was no cytotoxic effects on NIH3T3 and HeLa cells up to 40% of extract concentrations as determined by concerned assays.

Metoprolol succinate [24] (Drug Bank ID: DB00264) was selected as model drug. Metoprolol succinate is a *β*1 adreno-receptor antagonist generally used in angina-pectoris and hypertension. It has an oral bioavailability of 50%. It undergoes hepatic metabolism, and its elimination half-life is 3-7 hours. Therefore, it is suitable candidate for the design of bioadhesive drug delivery systems.

In the present investigation, multiparticulate system for intestinal delivery of metoprolol succinate based on SPHC was attempted. Such system provides an intimate contact for longer duration between the drug delivery system and mucus layer of intestine. This might be helpful in prolongation of drug release, increased absorption, bioavailability enhancement, and reducing the frequency of administration of the drug.

## 2. Experimental

### 2.1. Materials

Metoprolol succinate, acrylic acid, chitosan, Xanthan gum, and HPMC were purchased from Yarrow Chem., Mumbai, India. N,N′-Methylenebisacrylamide, and N,N,N′,N′-tetramethylethylenediamine were purchased from Loba Chemie Pvt. Ltd., Mumbai, India. Sodium alginate and ammonium persulfate were purchased from Finar Chemicals Limited, Ahmedabad, India. Span 80 was purchased from Chemdyes Corporation, Mumbai, India. Double distilled water (DDW), 0.1 N HCL and phosphate buffer pH 6.8 were prepared in laboratory. All other chemicals used were of analytical grade and used as obtained.

### 2.2. Preparation of SPHC Particles

SPHCs were prepared by gas blowing method [1]. Chitosan, Xanthan gum, and sodium alginate were used as bioadhesive polymers. To control the drug release up to 10 hours, release retardant polymers, namely, HPMC K4M, HPMC K15M, and HPMC K100M, with different concentration, were studied. Acrylic acid, bioadhesive polymer, release retardant polymer, methylene-bis-acrylamide, tetramethylethylenediamine, span 80, and DDW were added subsequently into a test tube at room temperature to prepare SPHCs. Ammonium persulfate was added to the reaction mixture after adjustment of the pH to 5 with 5 M NaOH solution. Reaction mixture was shaken vigorously for 10 minutes to complete the polymerization reaction. NaHCO_3_ was added very quickly to the mixture at last, which leads to the SPHCs formation. SPHCs were dried at 50°C for 24 hours. SPHCs were crushed by grinding method and passed through appropriate sieve to get uniform sized particles of about 500 *μ*m. SPHCs particles were stored in airtight container until further use.  Table 1 shows the composition of SPHCs for preliminary screening containing different bioadhesive and release retardant polymers. Batches from P1 to P3 were prepared to select the best suited bioadhesive polymers. After selection of bioadhesive polymer from batches P1 to P3, P4 to P9 batches were prepared to select suitable release retardant polymer. Percentage bioadhesion, *in vitro* drug release, and drug content were used as selection parameters to select effective bioadhesive polymer and release retardant polymer.

### 2.3. Scanning Electron Microscopy Analysis

The dried SPHCs were cut to expose their internal structure for SEM study, while SPHC particles were analyzed directly. The morphology and porous structure of dried SPHCs and particles thereof were analyzed by using JEOL JSM-5610 Scanning Electron Microscope (JEOL Worldwide, India) with an operating voltage of 10 kV.

### 2.4. Swelling Ratio, Density, and Porosity Measurement of SPHC Particles

The dried SPHC particles were immersed in an excess of DDW for swelling study. At regular time intervals, the SPHC particles were removed from the medium and reweighed to determine *M*_*s*_. The equilibrium swelling ratio was calculated from

(1) $$\begin{array}{c} Q=\frac{\left(M_{s}-M_{d}\right)}{M_{d}}, \end{array}$$

where *Q* is equilibrium swelling ratio *M*_*s*_ and *M*_*d*_ are mass in swollen and dried states, respectively.

The apparent density of the dried SPHC particles was measured using the solvent displacement method. SPHC particles with known mass were immersed in a predetermined volume of hexane in a graduated cylinder. The volume of hexane displaced by SPHC particles was measured. The apparent density was calculated using

(2) $$\begin{array}{c} \text{Density}=\frac{M}{V}, \end{array}$$

where *V* is the volume of hexane displaced by SPHC particles and *M* is the mass of SPHC particles.

The porosity of dried SPHC particles was determined from pore volume and bulk volume, by immersing a definite quantity of SPHC particles in hexane overnight. After removing the excess solvent, the SPHC particles were reweighed. The porosity was calculated from (3).

(3) $$\begin{array}{c} \text{Porosity}=\frac{\left(w-\;w_{0}\right)}{\rho V_{T}}, \end{array}$$

where *w*_0_ and *w* are the weights of SPHC particles before and after immersion, *ρ* is the density of solvent, and *V*_*T*_ is the total volume of SPHC particles.

### 2.5. *In Vitro* Washoff Test for Mucoadhesion

SPHC particles were evaluated for mucoadhesive property by an *in vitro *washoff test method [19]. Freshly excised pieces of intestinal mucosa of 3 cm^2^ from rat (Protocol no. SSPC/IAEC/15/03/2012) were mounted onto glass slides (3 × 1 inch) with cyanoacrylate glue. Approximately 50 SPHC particles were spread onto each wet rinsed tissue specimen, and immediately thereafter the support was hung onto the arm of a USP tablet disintegrating test apparatus (Electrolab, Mumbai, India). The disintegrating test apparatus was operated in such a manner so that the tissue specimen showed a slow, regular up and down movement in phosphate buffer, pH 6.8 at 37°C contained in a 1 L vessel. At the regular interval of 1 hour, the machine was stopped and the number of SPHC particles still adhering to the tissue was counted up to 8 hours.

### 2.6. Preparation of SPHC Particles Based Drug Delivery System

Accurately weighed 100 mg of metoprolol succinate was dissolved in 10 mL of DDW. 200 mg of SPHC particles were kept in the drug solution for 12 hours. The drug loaded SPHC particles were filtered and dried in hot air oven at 50°C for 12 hours. These drug loaded SPHC particles were filled in hard gelatin capsule (00). The capsules were coated using CAP as an enteric coating polymer by dip coating method. The capsules were dipped alternatively in CAP solutions (3–6% w/v) prepared in acetone and dried until an expected weight gain of 8–12%. Sudan IV was used as coloring agent. Enteric coated capsules should resist disintegration in 0.1 N HCL for minimum of 2 hours.

### 2.7. FTIR Spectroscopy

FTIR spectra of pure drug, SPHC particles, and drug loaded SPHC particles were recorded using KBr mixing method on FTIR spectrophotometer (FTIR-8400S, Shimadzu, Japan) in the range of 4000–400 cm^−1^ to study the interaction between drug and excipients used.

### 2.8. Differential Scanning Calorimetry (DSC)

To characterize the thermal behavior of the drug and drug loaded SPHC particles, DSC thermograms were recorded using a differential scanning calorimeter (DSC-60, Shimadzu, Japan). At a constant nitrogen flow rate of 40 mL/min, samples were heated at a linear heating rate of 10°C/min between 30 and 300°C.

### 2.9. Optimization Using Full Factorial Design

A 3^2^ randomized full factorial design was adopted where two independent variables, namely, amount of chitosan (*X*_1_) and amount of HPMC K4M (*X*_2_) were selected, and each was evaluated at three levels. Drug loading, cumulative percentage drug release at 3 hours (Q3), 6 hours (Q6), and 9 hours (Q9), and percentage bioadhesion were chosen as dependent variables. The optimized batch was evaluated for swelling ratio, percentage porosity, and density. The formulation layout for the factorial batches (F1–F9) is shown in Table 2.

### 2.10. Drug Content Determination

Accurately weighed 100 mg of drug loaded SPHC particles were crushed in mortar using pestle and were suspended in 25 mL phosphate buffer pH 6.8 for overnight. The solution was filtered, and after suitable dilution, the absorbance was measured at 222 nm using a double-beam UV-Visible spectrophotometer (UV-1800, Shimadzu, Japan). The drug content was calculated using calibration curve equation.

### 2.11. *In Vitro* Drug Release Study

The release rate of metoprolol succinate from prepared drug delivery system (*n* = 3) was determined using USP XXIV dissolution test apparatus II (paddle method). The dissolution test was performed at 50 rpm using 900 mL 0.1 N HCl for the first 2 hours and then in phosphate buffer pH 6.8 for 8 hours at 37 ± 0.5°C. Ten mL of samples were withdrawn at predetermined interval times (hourly) for 10 hours from the dissolution medium and were replaced immediately with fresh medium. The samples were analyzed using UV-Visible spectrophotometer (UV-1800, Shimadzu, Japan) at respective *λ*_max⁡_. The dissolution profiles of all factorial batches were fitted to various models [25], namely, zero order, first order, Higuchi [26], Hixson and Crowell [27], and Korsmeyer et al. [28] as shown in Table 3 to ascertain the release kinetic of drug. The method described by Korsmeyer et al. was used to understand drug release mechanism. Response surface and contour plots were used for better understanding of the optimized amounts of chitosan and HPMC in the formulations.

### 2.12. Stability Study

The optimized formulation was kept in airtight containers and stored in the stability chamber (TH-90S, Thermolab, India) 40 ± 2°C/75 ± 5% RH for 6 months. Results of *in vitro *drug release studies obtained after and before six months were compared. At the end of study, samples were analyzed for the percentage drug content, *in vitro* drug release, and percentage bioadhesion. Comparison of both batches was carried out using similarity factor (*f*_2_) calculated from

(4) $$\begin{array}{c} f_{2}=50\times \log\left\{{\left[1+\left(\frac{1}{n}\right)\sum_{t=1}^{n}{\left(R_{t}-T_{t}\right)}^{2}\right]}^{-0.5}\times 100\right\}, \end{array}$$

where *n* is the number of dissolution time points and *Rj* and *Tj* are the percentages dissolved of the reference product and test product at each time point *j*, respectively.

## 3. Results and Discussion

### 3.1. Preparation of SPHC Particles

Homogeneous SPHCs were prepared by using gas blowing method. Acrylic acid, methylene-bis-acrylamide, ammonium persulfate, tetramethylethylenediamine, Span 80, and sodium bicarbonate were used as monomer, cross-linker, polymerization initiator, catalyst, foam stabilizer, and foaming agent, respectively. SPHC preparation was influenced by the pH of acrylic acid monomer solution so it was maintained at the pH 5 which provided the foam stability and the proper formation rate. The results of the drug loading, bioadhesion time, and *in vitro* drug release of preliminary trials are shown in Table 4. The prepared SPHC particles should remain adhered to the intestinal mucosa for 8 hours. Batch P1 containing 1% w/v chitosan as a bioadhesive polymer showed bioadhesion up to 8 hours and good drug loading compared to batches P2 and P3. Batch P1 achieved the bioadhesion up to 8 hours but the drug was not released within targeted 8 hours, so release retardant polymers, namely, HPMC K4M, K15M and K100M with different concentrations were incorporated in batches P4 to P9. Drug loading and *in vitro* drug release were varied as release retardant polymer was changed. Batches P6 and P9 contain HPMC K100M, which is higher viscosity grade, were failed to prepare SPHC. Batch P3 with 1% w/v HPMC K4M showed good drug loading, but it could control the drug release only for 7 hour. Batches P5 and P8 with 1-2% w/v HPMC K15M could control the drug release for 10 hours, but they showed less drug loading compared to batch P7. Batch P7 with 2% w/v HPMC K4M could control drug release up to 10 hours, with high drug loading. From the preliminary studies 1% w/v chitosan and 2% w/v HPMC K4M were selected as bioadhesive and release retardant polymers, respectively.

### 3.2. Scanning Electron Microscopy Analysis

**Figure 1:**
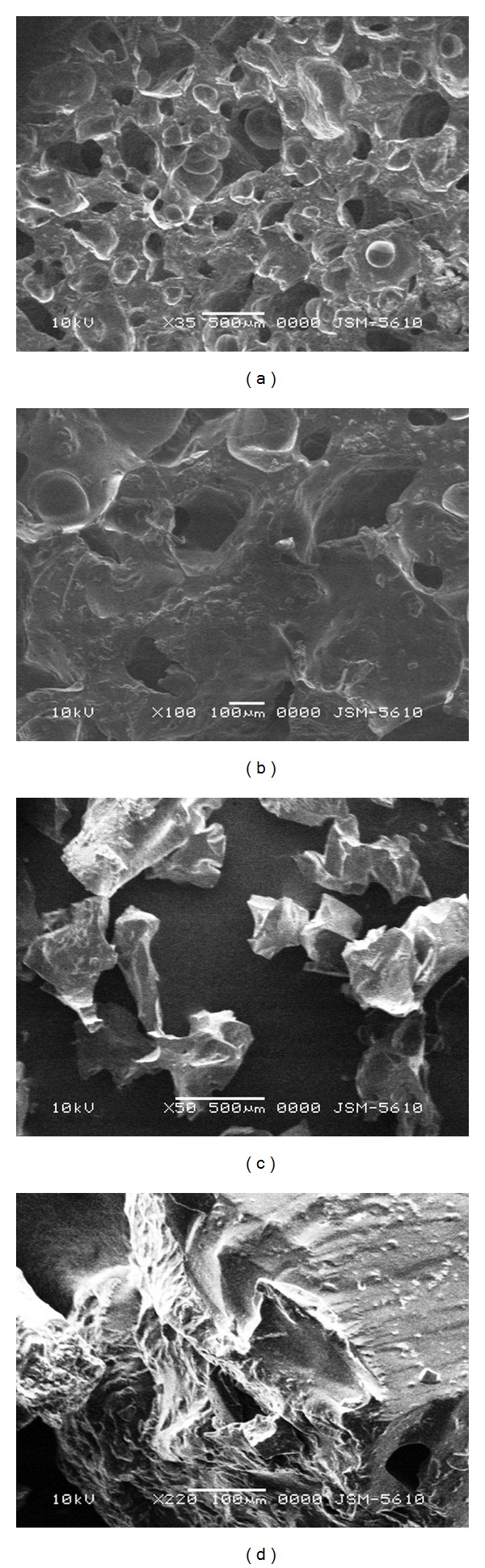
SEM pictures of SPHC and SPHC particles. (a) SPHC, ×35 magnification; (b) SPHC, ×50 magnification; (c) SPHC particles, ×35 magnification; (d) SPHC particles, ×50 magnification.

Figure 1 shows the SEM pictures of SPHC and SPHC particles. SPHC possessed numerous pores and retained its superporous structure. As shown in Figures 1(c) and 1(d), the SPHC particles showed the effect of compression on its surface. The destruction of superporous structure is observed at many places that might be due to their conversion by grinding method, however, few pores are not disturbed and visible.

### 3.3. Swelling Study, Density, and Porosity Measurement of SPHC Particles

Swelling study, apparent density, and percentage porosity study were carried out for batch P5, which was selected as a prototype formula for further study, without drug. SPHC particles showed good swelling ratio of 87.58 ± 5.96%, porosity of 47.11 ± 1.80%, and apparent density of 0.48 ± 0.17. SPHC particles showed higher swelling ratio and percentage porosity than ordinary conventional hydrogels. SPHC particles showed more than 80% drug loading.

### 3.4. Preparation of SPHC Particles Based Drug Delivery System

SPHC particles based drug delivery system was in the form of microparticles filled in capsule and was coated using CAP as an enteric coating polymer by dip coating method. As the concentration of CAP was increased, capsule integrity was also improved. CAP concentrations of 3 and 4% w/v failed to provide intactness to capsule for 2 hour. 5% w/v CAP could keep the capsule intact for more than 2 hours so it was selected for coating which can resist disintegration in 0.1 N HCL.

### 3.5. FTIR Spectroscopy

**Figure 2:**
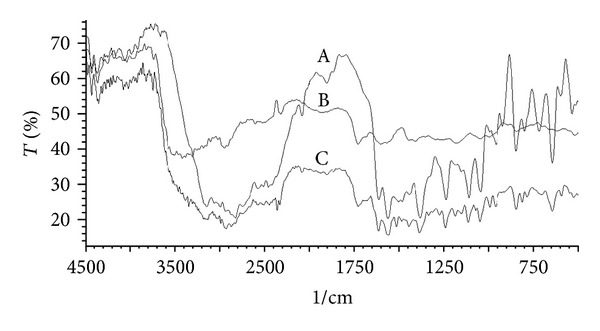
FTIR spectra of (A) metoprolol succinate; (B) SPHC particles; (C) metoprolol succinate loaded SPHC particles.

Figure 2 shows the FTIR spectra of metoprolol succinate, SPHC, and drug loaded SPHC, respectively. The characteristic peaks of secondary alcohol (3350 cm^−1^), secondary amine (3136 cm^−1^), and methyl ether (1114 cm^−1^) of metoprolol succinate that are not shifted significantly in metoprolol succinate loaded SPHC particles revealed that there is no interaction between drug and SPHC ingredients present in formulations.

### 3.6. DSC Study

**Figure 3:**
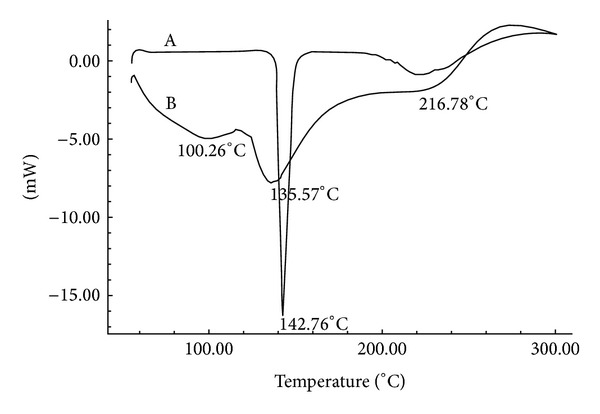
DSC thermograms of (A) metoprolol succinate; (B) metoprolol succinate loaded SPHC particles.

DSC thermograms of pure metoprolol succinate and metoprolol succinate loaded SPHC particles are shown in Figure 3. Pure powdered metoprolol succinate showed a single sharp melting endotherm at 142.76°C corresponding to its melting, and a single peak indicates that the drug sample is free from impurities. DSC thermogram of crushed and powdered drug loaded SPHC particles shows the broadened low intense melting peak shifted toward lower temperature at 135.57°C. This might be due to the conversion of crystalline state to amorphous state as the drug was incorporated into SPHC particles by soaking method. This study revealed that the drug was pure and compatible with SPHC particles with no major interactions.

### 3.7. Drug Content, Swelling Ratio, Porosity, and Density of Factorial Batches

The drug content, swelling ratio, porosity, and density of factorial batches are shown in Table 5. The results show that percentage drug content and swelling ratio of factorial batches decreased as the amount of HPMC K4M increased. SPHC particles are found to be porous and low dense as they showed percentage porosity of more than 35 and density less than 0.75 g/cc, respectively. Drug contents were found to be dependent on the porosity, as reflected in Table 5. As the porosity of SPHC particles increases, the drug content decreases. It might be possible that highly porous structure provides less volume of solid hydrogel (*V*_*T*_ − *V*_*P*_) in SPHC particles and hence less amount of drug diffused in this solid part.

### 3.8. Bioadhesion Study of Factorial Batches

**Figure 4:**
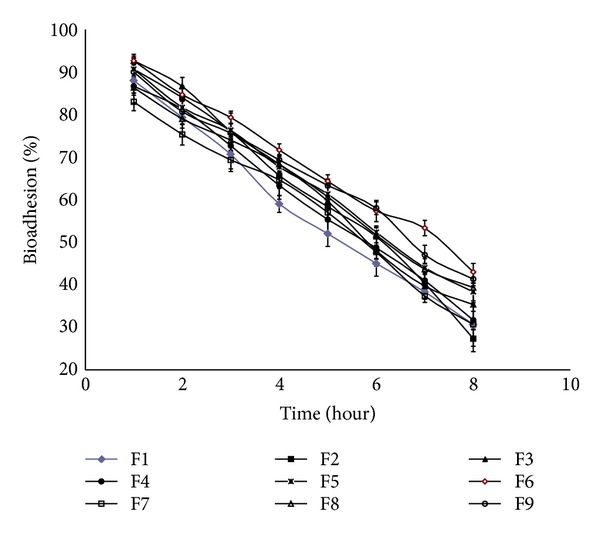
Percentage bioadhesion of factorial batches.

Figure 4 shows percentage bioadhesion of factorial batches. The results shows that as the amount of chitosan increased the percentage bioadhesion of SPHC particles increased. Batch F6 is found to be more bioadhesive to mucosal surface as 43% of SPHC particles remain adhered to mucosa up to 8 hours. All formulations showed less % bioadhesion that was less than 50% of initial one. However, it could be possible that as SPHC particle detached from the mucosa to it still possesses the tendency to adhere newer surface of intestinal mucosa. As the time passed the percentage bioadhesion decreased, however, the SPHC particles could adhere to the other part of the long intestine, so the drug release was considered to be maintained for 8 hour.

### 3.9. *In Vitro* Drug Release Study of Factorial Batches

**Figure 5:**
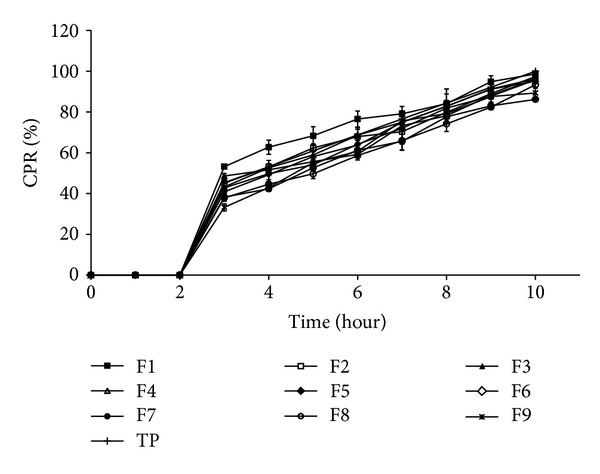
Cumulative percentage drug release (CPR%) of factorial batches.

**Figure 6:**
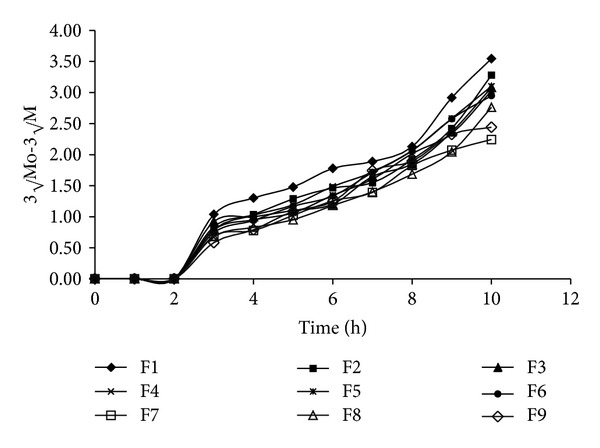
Hixson-Crowell cube root plots of factorial batches.

**Figure 7:**
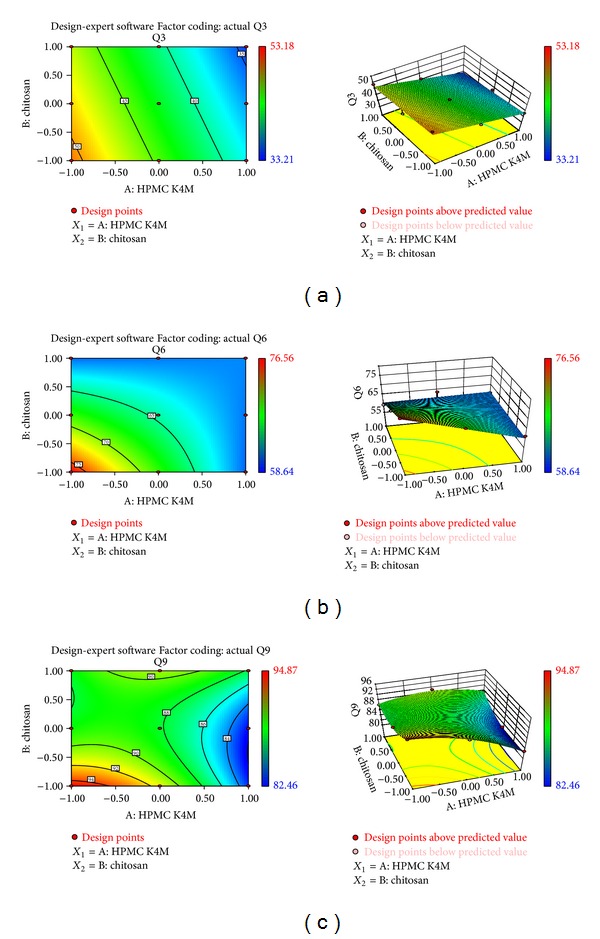
(a) Response surface plot and contour surface plot for cumulative percentage drug release at 3 hours (Q3). (b) Response surface plot and contour surface plot for cumulative percentage drug release at 6 hours (Q6). (c) Response surface plot and contour surface plot for cumulative percentage drug release at 9 hours (Q9).

Figure 5 shows drug release profiles of factorial batches. All factorial batches showed drug release up to 10 hours. Increased amount of HPMC K4M and chitosan decreased the drug release.  Table 6 shows the release rate constants calculated from the slope of the appropriate plots and the determined regression coefficients (*r* ^2^). The *in vitro* drug release of batch F6 is best explained by Hixson-Crowell model as the plots showed the highest linearity (*r* ^2^ = 0.9786) indicating that the drug release follows Hixson-Crowell cube root law, followed by first order (*r* ^2^ = 0.9753), zero order (*r* ^2^ = 0.9404), and Higuchi (*r* ^2^ = 0.8823). The corresponding Hixson-Crowell plots are shown in Figure 6. The Korsmeyer-Peppas model indicated a good linearity (*r* ^2^ = 0.9956) and the value of release exponent *n* which is 0.7246 indicates anomalous nonfickian transport drug release, which appeared to indicate a coupling of the diffusion and polymer relaxation mechanisms. It indicates that the drug release from SPHC particles could be controlled by more than one process. Batch F6 was optimized as it showed desired drug release profile, bioadhesion, and drug content. Response surface and contour plots as shown in Figure 7 for drug release at 3, 6, and 9 hours support the selection of optimized amounts of chitosan and HPMC.

### 3.10. Stability Study

The optimized formulation, batch F6, stored at 40 ± 2°C/75 ± 5% RH was found to be stable for 6 months. Drug release profiles of optimized formulation before and at the end of study were similar (*f*_2_ = 72.07). Study indicates that after storage drug release, bioadhesion and drug content were found to be nearly similar.

## 4. Conclusions

The present investigation showed the development of SPHC particles as multiparticulate bioadhesive system with high drug loading. SEM studies showed the formation of interconnected pores in SPHC, which were not affected significantly after conversion to particles. FT-IR and DSC study showed that the drug was compatible with SPHC particles. SPHC particles were low dense, porous, and having good swelling capacity. On the basis of preliminary trials, chitosan and HPMC K4M were selected as bioadhesive and release retardant polymers, respectively. Batch F6 containing 300 *μ*L of chitosan (1% w/v) and 200 *μ*L of HPMC K4M (2% w/v) showed desired drug release, bioadhesion, and drug content. Drug release from the prepared system follows Hixson-Crowell cube root law and anomalous nonfickian transport. Accelerated stability testing showed that the drug delivery system was stable as per ICH guidelines. The bioadhesive SPHC particles based drug delivery system for an intestinal delivery of metoprolol succinate has been successfully prepared. This system can provide a suitable platform for drug delivery systems based on SPHC particles and open a new insight into intestinal delivery.

## Figures and Tables

**Table 1:** Composition of SPHCs for preliminary screening of polymers.

Ingredients	P1	P2	P3	P4	P5	P6	P7	P8	P9
AA* (50% v/v)	500 *μ*L	500 *μ*L	500 *μ*L	500 *μ*L	500 *μ*L	500 *μ*L	500 *μ*L	500 *μ*L	500 *μ*L
Chitosan (1% w/v)	200 *μ*L	—	—	200 *μ*L	200 *μ*L	200 *μ*L	200 *μ*L	200 *μ*L	200 *μ*L
Xanthan gum (1% w/v)	—	200 *μ*L	—	—	—	—	—	—	—
Sodium alginate (1% w/v)	—	—	200 *μ*L	—	—	—	—	—	—
HPMC^†^ K4M (1% w/v)	—	—	—	200 *μ*L	—	—	—	—	—
HPMC^†^ K15M (1% w/v)	—	—	—	—	200 *μ*L	—	—	—	—
HPMC^†^ K100M (1% w/v)	—	—	—	—	—	200 *μ*L	—	—	—
HPMC^†^ K4M (2% w/v)	—	—	—	—	—	—	200 *μ*L	—	—
HPMC^†^ K15M (2% w/v)	—	—	—	—	—	—	—	200 *μ*L	—
HPMC^†^ K100M (2% w/v)	—	—	—	—	—	—	—	—	200 *μ*L
BIS^‡^ (2.5% w/v)	100 *μ*L	100 *μ*L	100 *μ*L	100 *μ*L	100 *μ*L	100 *μ*L	100 *μ*L	100 *μ*L	100 *μ*L
TEMED^§^ (20% v/v)	25 *μ*L	25 *μ*L	25 *μ*L	25 *μ*L	25 *μ*L	25 *μ*L	25 *μ*L	25 *μ*L	25 *μ*L
APS^||^ (20% w/v)	25 *μ*L	25 *μ*L	25 *μ*L	25 *μ*L	25 *μ*L	25 *μ*L	25 *μ*L	25 *μ*L	25 *μ*L
Span 80 (10% v/v)	30 *μ*L	30 *μ*L	30 *μ*L	30 *μ*L	30 *μ*L	30 *μ*L	30 *μ*L	30 *μ*L	30 *μ*L
DDW^¶^	300 *μ*L	300 *μ*L	300 *μ*L	300 *μ*L	300 *μ*L	300 *μ*L	300 *μ*L	300 *μ*L	300 *μ*L
Sodium bicarbonate	200 mg	200 mg	200 mg	200 mg	200 mg	200 mg	200 mg	200 mg	200 mg

*AA: acrylic acid; ^†^HPMC: hydroxypropylmethyl cellulose; ^‡^BIS: N,N′-methylene-bis-acrylamide; ^§^TEMED: N,N,N′,N′-tetramethylethylenediamine; ^||^APS: ammonium persulfate; ^¶^DDW: double distilled water.

**Table 2:** Coding and composition of factorial batches.

	F1	F2	F3	F4	F5	F6	F7	F8	F9
Coding of variables									
*X*_1_ level	−1	0	+1	−1	0	+1	−1	0	+1
*X*_2_ level	−1	−1	−1	0	0	0	+1	+1	+1
Ingredients									
AA* (50% v/v) (*μ*L)	500	500	500	500	500	500	500	500	500
Chitosan (1% w/v) (*μ*L)	100	200	300	100	200	300	100	200	300
HPMC^†^ K4M (2% w/v) (*μ*L)	100	100	100	200	200	200	300	300	300
BIS^‡^ (2.5% w/v) (*μ*L)	100	100	100	100	100	100	100	100	100
TEMED^§^ (20% v/v) (*μ*L)	25	25	25	25	25	25	25	25	25
APS^||^ (20% w/v) (*μ*L)	25	25	25	25	25	25	25	25	25
Span 80 (10% v/v) (*μ*L)	30	30	30	30	30	30	30	30	30
DDW^¶^ (*μ*L)	300	300	300	300	300	300	300	300	300
Sodium bicarbonate (mg)	200	200	200	200	200	200	200	200	200

*AA: acrylic acid; ^†^HPMC: hydroxypropylmethyl cellulose; ^‡^BIS: N,N′-methylene-bis-acrylamide; ^§^TEMED: N,N,N′,N′-tetramethylethylenediamine; ^||^APS: ammonium persulfate; ^¶^DDW: double distilled water.

**Table 3:** Models to ascertain the kinetic of drug release.

Mathematical model	Equation
Zero order	*Q*_*t*_ = *Q*_0_ + *K*_0_ *t*
First order	ln⁡*Q*_*t*_ = ln⁡*Q*_0_ + *K*_1_ *t*
Higuchi	*Q*_*t*_ = *K*_*H*_ *t* ^1/2^
Hixson-Crowell	*Q*_0_^1/3^ − *Q*_*t*_^1/3^ = *K*_*s*_ *t*
Korsmeyer-Peppas	*Q*_*t*_/*Q*_*∞*_ = *K*_*k*_ *t*_*n*_

*Q*_*t*_: amount of drug released in time *t*; *Q*_0_: initial amount of drug in the dosage form; *Q*_*∞*_: total amount of drug dissolved when the dosage form is exhausted; *K*_0_, *K*_1_, *K*_*H*_, *K*_*s*_, *K*_*k*_: release rate constants; *n*: release exponent (indicative of drug release mechanism).

**Table 4:** Results of preliminary trials.

Parameters	P1	P2	P3	P4	P5	P6	P7	P8	P9
Drug loading (%)	91.77 ± 3.45	82.46 ± 3.72	89.74 ± 4.70	88.24 ± 2.87	84.40 ± 3.88	SPHC was not formed	74.53 ± 2.42	70.93 ± 2.08	SPHC was not formed
Bioadhesion time (h)	8	7	5	8	8		8	8	
CPR* (%)	99.10	100.84	100.20	98.03	94.98		87.22	81.24	
*t*_100%_^†^ (h)	2	3	2	5	8		8	8	

*CPR: cumulative percentage drug release; ^†^ *t*_100%_: time required for 100% drug release.

**Table 5:** Drug content, swelling ratio, porosity, and density of factorial batches.

Batch code	Drug content*	Swelling ratio^∗,†^	Porosity^∗,†^	Density (g/cc)^∗,†^
F1	91.31 ± 1.79	122.11 ± 14.29	37.10 ± 4.41	0.57 ± 0.07
F2	90.53 ± 2.22	112.95 ± 13.45	42.74 ± 4.51	0.61 ± 0.08
F3	86.52 ± 3.69	099.64 ± 12.72	41.17 ± 3.52	0.69 ± 0.02
F4	88.51 ± 2.38	111.14 ± 11.76	39.92 ± 6.22	0.70 ± 0.02
F5	84.81 ± 1.89	095.34 ± 08.15	36.87 ± 1.99	0.65 ± 0.08
F6	84.23 ± 2.74	093.66 ± 12.43	42.62 ± 2.67	0.62 ± 0.03
F7	81.47 ± 2.06	097.31 ± 06.60	47.96 ± 4.49	0.75 ± 0.05
F8	76.52 ± 2.30	085.62 ± 05.94	44.34 ± 2.29	0.68 ± 0.07
F9	75.14 ± 1.65	072.54 ± 03.83	51.92 ± 3.69	0.70 ± 0.06

*Mean ± SD, *n* = 3.

^†^SPHC particles without drug loading.

**Table 6:** Parameters and determination coefficients of the linearization of SPHC particles based drug delivery systems.

Model		F1	F2	F3	F4	F5	F6	F7	F8	F9
Zero order	*K*_0_	10.8270	10.4408	10.2299	10.6811	10.4441	10.6516	9.7257	9.9020	10.3401
	*r* ^2^	0.8775	0.9175	0.9171	0.9294	0.9374	0.9404	0.9396	0.9502	0.9498
First order	*K*_1_	0.0839	0.1043	0.1034	0.1128	0.1172	0.1217	0.1226	0.1274	0.1405
	*r* ^2^	0.9708	0.9796	0.9795	0.9764	0.9898	0.9753	0.9681	0.9963	0.9298
Higuchi	*K*_*H*_	37.3735	35.4702	34.6312	36.1576	35.1743	35.8583	32.7297	33.0886	34.5900
	*r* ^2^	0.8656	0.8767	0.8702	0.8817	0.8803	0.8823	0.8810	0.8784	0.8800
Hixson-Crowell	*K*_*s*_	0.3626	0.3246	0.3089	0.3314	0.3152	0.3251	0.2572	0.2795	0.2897
	*r* ^2^	0.9475	0.9407	0.9381	0.9739	0.9653	0.9786	0.9789	0.9546	0.9861
Korsmeyer-Peppas	*n*	0.4991	0.6165	0.5924	0.6720	0.6886	0.7246	0.7288	0.7464	0.8528
	*r* ^2^	0.9883	0.9845	0.9254	0.9977	0.9844	0.9956	0.9855	0.9841	0.9857

*K*_0_, *K*_1_, *K*_*H*_, *K*_*s*_: release rate constants; *n*: release exponent.
